# Urogenital schistosomiasis outbreak in a basic school, Volta Region, Ghana: a case-control study

**DOI:** 10.11604/pamj.2022.43.191.33362

**Published:** 2022-12-13

**Authors:** Paul Henry Dsane-Aidoo, Magdalene Akos Odikro, Holy Alomatu, Desmond Ametepi, Peace Selagbe Akwensy, Donne Kofi Ameme, Ernest Kenu

**Affiliations:** 1 Ghana Field Epidemiology and Laboratory Training Program, School of Public Health, Legon, Ghana,; 2 Ghana Health Service, Ketu North District Health Directorate, Volta Region, Ghana,; 3 Department of Epidemiology, School of Public Health, University of Ghana, Legon, Ghana

**Keywords:** *Schistosoma haematobium*, case-control studies, Ghana, disease outbreaks, mass drug administration

## Abstract

**Introduction:**

schistosomiasis is a neglected parasitic infection caused by nematode worms. It affects approximately 200 million people globally. Prevalence in Ghana is 23.3%, mostly affecting school children. On November 28^th^ 2018, the Disease Surveillance Department received reports of increase in occurrence of bloody urine among students of a basic school in the Volta Region. We investigated to identify the agent and source, to determine the magnitude, risk factors and to implement control measures.

**Methods:**

we conducted a case-control study. A suspected case was any student of the school, who has bloody urine with or without: dysuria, itching of the skin, frequent urination or lower abdominal pain from September 2018 to November 2018. A confirmed case was one with laboratory-isolation of Schistosoma ova in appropriate urine sample. We identified cases from the school and hospital records. We collected socio-demographic, clinical and exposure data from cases and controls. Descriptive and inferential analysis were performed to estimate odds ratios at 95% confidence intervals (CI) to determine associations.

**Results:**

of 880 students, 112 suspected cases were identified (attack rate = 12.7%). Mean age of suspected cases was 14-years (standard deviation = ±3.5). Confirmed cases were 76.8%(86/112). Males had twice odds of becoming cases (cOR = 2.3, 95% CI = 1.35-3.96). Fishing (cOR = 7.29, 95% CI = 4.08-13.04) and swimming (aOR = 44.63, 95% CI = 4.73-420.86) were factors significantly associated with infection. Students with previous history of bloody urine had greater odds of being cases (aOR = 47.9, 95% CI = 4.19-546.55).

**Conclusion:**

Schistosoma haematobium was isolated in this outbreak. Fishing and swimming were risky water-related activities. WASH education and mass drug administration with Praziquantel were control measures.

## Introduction

Schistosomiasis is a neglected parasitic infection caused by a trematode worm of genus *Schistosoma*. Humans are the definitive hosts for the infection, and freshwater snails are intermediate hosts. The snails release infective forms of the organism called cercariae that may penetrate the skin of individuals exposed to the water body [1,2]. The disease affects approximately 200 million people globally [3] with about 90% of infections in Africa. About 78 countries mostly in Africa, Eastern Asia, and South America have reported Schistosomiasis [4]. Ghana's countrywide prevalence is estimated at 23.3%, with a localized prevalence level of >50% [5].

There are two forms of the disease among humans in Ghana; Urogenital Schistosomiasis caused by *Schistosoma haematobium*, and Intestinal Schistosomiasis caused by *Schistosoma mansoni* [6,7]. Urogenital Schistosomiasis affects the urinary and the genital systems. A key sign of urogenital schistosomiasis is haematuria or blood in urine, but among females may present as vaginal discharges or bleeding [2]. It may be acute [8] or chronic leading to bladder cancers, progressive damage to gastrointestinal organs, infertility [4,6], and in some cases death [3]. Poor communities with inadequate water supply and populations that engage in recreational, occupational, and domestic use of dams and freshwater bodies are mostly affected [9]. Climate change, living close to water bodies, engagement in irrigation, and construction of dams are significant risk factors [10]. Children of school-going age are most vulnerable [9], usually suffering from anemia, stunting, and poor learning abilities. In Ghana, occurrences of Urogenital Schistosomiasis have been investigated among children of school-going age. Mass drug administration has been the key intervention [7,11,12].

On November 28, 2018, the Disease Surveillance Department of the Ghana Health Service received reports of an increase in bloody urine among students of a basic school in the Ketu North District of the Volta Region of Ghana. The school is located within a farming and fishing community that has two rivers as the water source. This was the first reported cluster of cases within the District in 2018. We investigated the outbreak to determine the magnitude and risk factors and to implement control measures.

## Methods

**Study design:** we conducted an unmatched case-control study among school children.

**Study setting:** the outbreak occurred at a basic school in the Ketu North District of the Volta Region of Ghana. The school has 880 students who live in three communities, namely; Yia, Horme, and Dome. One Community Health Planning System (CHPS) compound serves these communities. A district hospital serves as the referral center for the communities. Farming and fishing are the main occupation of community members. The main water sources are two rivers A and B, which have been dammed for irrigation purposes. There has not been any report of clusters of bloody urine among school students in these three communities three years prior to this report.

**Stakeholder Engagements, Community Entry, Initial Data Review and Formulation of Case Definitions:** the outbreak investigation team met the District Health Authorities, the District Environmental Health Officers, teachers at the basic school, and Chiefs of the communities on November 29^th^, 2018. These meetings gave the team local permission to conduct the investigation, and helped obtain information about initial outbreak response.

We extracted district surveillance data on bloody urine from the District Health Information Management System (DHIMS-II). We determined the districts expected threshold from 2014 to 2018 using the moving average formula and plotted it against the cases of bloody urine reported over the period. This helped to confirm the outbreak. We reviewed medical records of affected students, the initial line-list, and formulated the outbreak case definition. The symptoms in the case definition were determined from literature review, and were selected based on their frequency of occurrence among the cases.

A suspected Schistosomiasis case was “any student of the basic school, who complains of bloody urine with or without one or more of the following: dysuria, itching of the skin, frequency of urination and lower abdominal pain from September 2018 to November 2018”. A confirmed schistosomiasis case was “any student from the basic school with laboratory isolation of Schistosoma ova from urine sample”.

**Case finding:** we performed an active case search for suspected cases in the school using the case definition, and reviewed outpatient data at the district hospital for missed cases. The team trained teachers of the school to understand the disease presentation. During school hours, teachers engaged students and identified cases from each class. We interviewed these students further to confirm symptoms of the disease and their frequencies among identified cases determined. We updated the line list using demographic, clinical, exposure and laboratory variables. We reviewed the clinical management notes at the district hospital.

**Environmental assessment:** we conducted observational assessment of the environments of the three communities (Yia, Horme and Dome). We visited their sources of portable water as well as the dams, and looked for the activities of children along the two dams. We examined the banks of the dams for snails.

**Hypothesis testing and epidemiological investigation:** considering the distribution of bloody urine among the students of the school, we made the following hypothesis;

***Alternative hypothesis:*** engaging in water related activities in Dam B is more likely to result in occurrence of bloody urine among students than in Dam A.

***Null hypothesis:*** engaging in water related activities in Dam A and Dam B will equally result in occurrence of bloody urine among students.

We conducted a 1: 2 unmatched case-control study to test the hypothesis and to determine the source of the outbreak. A control was another student of the school without symptoms of urogenital schistosomiasis. We developed a screening tool to identify cases and controls among the students. The Regional and District Health Authorities provided permission for the epidemiological study. We obtained consent for students 18 years and older. Assent was obtained from legal guardians of students less than 18 years to be enrolled. This was following detailed explanation of the study to them. Cases in the school who granted consent, or for whom accent was obtained were recruited for the study. Controls were identified from all classes and numbered. We conducted random selection of controls for each case by balloting. Using a structured questionnaire, we obtained demographic and risk factor variables from both cases and controls.

A student was considered exposed if there is the history of indulging in either of the two dams, or both, in any water contact activity. This includes, fetching for domestic use, washing in the river, bathing, swimming, fishing or fetching the water for irrigation purposes. All exposures in the range of a few hours up to habitual engagement in activities in the dam were all considered significant for infestation to occur [8]. Students who did not participate in these activities were classified as unexposed.

**Exclusion criteria:** we excluded controls who have had a recent intake of Praziquantel for bloody urine [13].

**Laboratory assessment:** we collected midday mid-stream urine samples of all suspected cases into coded sterile urine containers pre-labeled with names of students. Samples were transported in a cold box at 4-6°C within 4 hours to the district laboratory for immediate macroscopic and microscopic assessments. The collected urine samples were analyzed for haematuria using urine chemistry reagent strips. The results were recorded as positive (trace, +, ++, and +++) or negative. Ten milliliters of each urine sample was transferred into 15 ml centrifuge tube and centrifuged at 3000 rpm for 3 minutes. The supernatant was decanted until the 1 ml mark. Using a pasteur pipette, 50 μl of the sediment was placed on a grease-free glass slide and observed under the microscope. The presence of *Shistosoma haematobium* ova were determined microscopically using the ×10 and ×40 objective of an optical light microscope.

**Data analysis:** quantitative data were entered into Microsoft Excel for cleaning, and analyzed with Stata 15. Using descriptive statistics, we analyzed data to provide frequencies and proportions for categorical variables. Mean (± standard deviation) for numeric variables were computed. Cross tabulation was done to determine variables that differed significantly between cases and controls. Chi-Square associations were considered significant when p<0.05. Bivariate analysis for crude odds ratios (cOR) at 95% CI was done and variables with p<0.05 were included in the multivariate model. Logistic regression was done to estimate adjusted odds ratios (aOR) at 95% CI to determine independent risks associated with the occurrence of schistosomiasis.

**Ethical consideration:** this outbreak investigation was deemed a response to a public health emergency by the Ghana Health Service and hence did not require a formal review by Ethical Review Committees. We obtained permission from the National Disease Surveillance Department through the Ghana Field Epidemiology and Laboratory Training Program. Permission was obtained from the Volta Regional Health Authority, the Ketu North District Health Directorate, as well as the Regional and District Education Services to access local data during the investigation.

## Results

**Figure 1:**
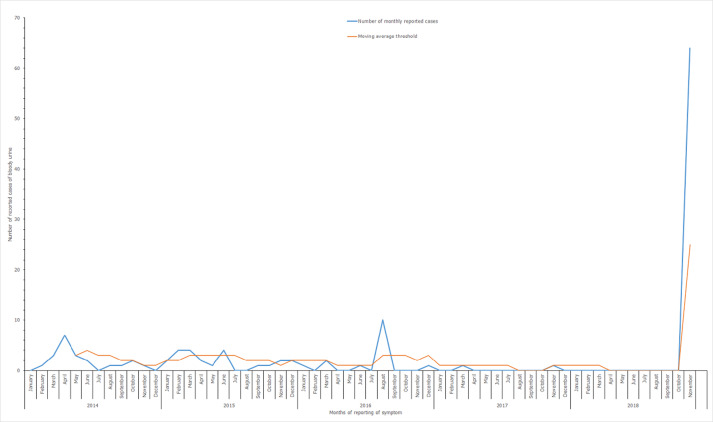
monthly distribution of bloody urine cases against moving average threshold for Ketu North District, 2014-2018

The moving average threshold analysis of bloody urine in Ketu North District showed potential outbreaks in 4 months within 2015 and 1 in August 2016 (Figure 1). The recent increase had recorded 64 cases of bloody urine before outbreak investigation started. This was demonstrated as a confirmed outbreak using the moving average curve in Figure 1.

**Demographic characteristics of suspected *schistosomiasis* cases:** of 880 students, 112 were reported to have had bloody urine (Table 1). Mean (SD) age of cases was 14 (±4) years. No deaths were recorded among students over the period of disease occurrence. Most cases lived in the Horrme community, 66% (74/112) (Table 1). All suspected cases reported haematuria (100%), with 39% (44/112) having bloody stool (Table 1). There was no identified case of vaginal discharge.

**Table 1:** socio-demography and symptoms of line listed schistosomiasis cases, the basic school, 2018

Factors	Number of cases	Proportion (%)
**Age category (N=112)**		
4-10	18	16.1
11-15	55	49.1
16-20	39	34.8
**Sex (N=112)**		
Female	40	35.7
Male	72	64.3
**Community (N=112)**		
Dome	17	15.2
Horme	74	66.1
Yia	21	18.7
**Class (N=112)**		
Pre-school	2	1.8
Primary School	40	35.7
Junior High School	70	62.5
**Symptoms (N=112)**		
Haematuria **(N=112)**	112	100
Dysuria **(N=112)**	92	82
Frequent urination **(N=112)**	88	78
Itchy skin **(N=112)**	86	77
Fever **(N=112)**	78	69
Lower abdominal pains **(N=112)**	72	64
Bloody stools **(N=112)**	44	39

**Attack rates:** the overall attack rate was 12.7% (112/880) and the sex-specific attack rate was 17.1% (72/420) for males. Junior high school had the highest class-specific attack rate of 39.8% (70/176). For clinical features, haematuria had the highest attack rate of 12.7% (112/880) among all students (Table 2).

**Table 2:** specific attack rates of suspected cases of schistosomiasis at the basic school, 2018

Category	No of suspected cases	Population at risk	Attack rate (%)
**Sex**			
Female	72	420	17.1
Male	40	480	8.3
**Class**			
Junior High School	70	176	39.8
Primary	40	462	8.7
Pre-schoolers	2	242	0.8
**Clinical Features**			
Haematuria	112	880	12.7
Dysuria	92	880	10.5
Frequent urination	88	880	10.0
Itchy skin	86	880	9.8
Fever	78	880	8.9
Lower abdominal pains	72	880	8.2
Bloody stools	44	880	5.0

**Figure 2:**
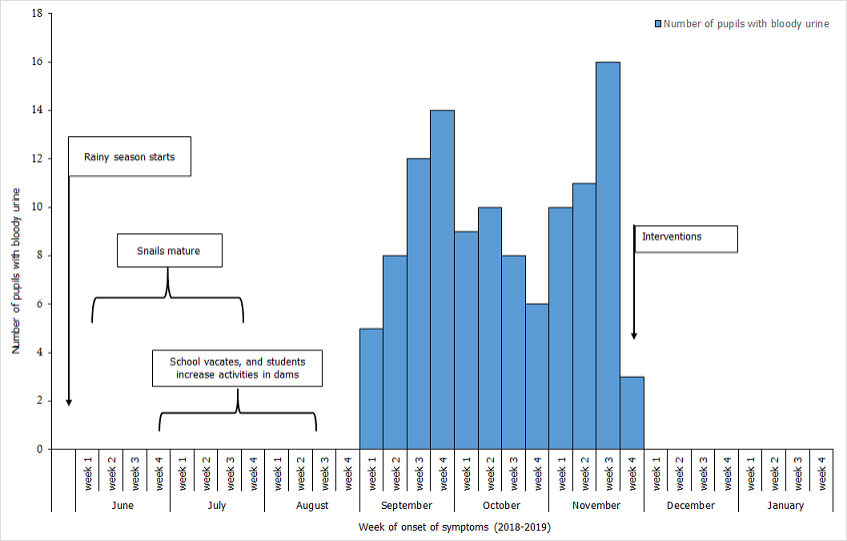
epidemiological curve of bloody urine among students of the basic school, Volta Region, 2018

**Epidemiological curve:** initial cases occurred in the first week of September 2018 and peaked in the third week of November 2018. Index cases reported in the first week of September 2018 with two peaks in week 4 of September and week 3 of November 2018. Following interventions, no new cases were recorded within the next two months (60 days) which covers more than 2 minimum incubation periods for schistosomiasis [8] (Figure 2).

**Environmental findings:** community members fetched water from dams for domestic purposes and farmers used dam water to irrigate farms. Students swam in the dams after school and participated in farming and fishing activities. Although community members confirmed the presence of fresh water snails, the investigative team could not identify snails at the banks of the dams.

**Laboratory findings:** overall, 58% (65/112) of suspected cases had frank blood in urine, 67.0% (75/112) had microscopically detectable red blood cells in urine. *Schistosoma haematobium* ova was isolated from 76.8% (86/112) urine samples. The ova was identified under wet microscopy mounts as having terminal spine.

**Inferential analysis for risk factors for bloody urine among students:** overall, 258 students were recruited in the analytical study involving 86 confirmed cases and 172 controls. Age category (χ^2^=0.1, p=0.982), unlike class category (χ^2^=6.5, p=0.037) did not differ significantly between cases and controls. Water sources and water-related activities that differed significantly among cases and controls included fishing (χ^2^=49.8, p<0.001), the dam fishing was done in (χ^2^=4.6, p<001), swimming (χ^2^=81.1, p<0.001), dam swimming is done in (χ^2^=4.8, p=0.028). Although engaging in farming by itself was not significant (χ^2^=0.3, p=0.723), the water source students used for irrigation of farms was significantly different (χ^2^=8.2, p=0.004) (Table 3).

**Table 3:** water based activities engaged in by cases and controls among students of the basic school, 2018

Characteristic and activity	Cases n (%)	Controls n (%)	χ^2^	p-value
**Age category**				
4-10	13 (15.1)	26 (15.1)	0.1	0.982
11-15	39 (45.4)	80 (46. 5)		
16-20	34 (39. 5)	66 (38.4)		
**Class category**				
Pre-school	4 (4.7)	8 (4.65)	6. 5	0.037*
Primary School	26 (30.2)	80 (46.5)		
Junior High School	56 (65.1)	84 (48.8)		
**Sex**				
Female	29 (33.7)	93 (54.1)	9. 5	0.002*
Male	57 (66.3)	79 (4.93)		
**History of bloody urine?**				
Previously experienced	49 (59.8)	30 (18.1)	43.9	<0.001*
Not previously experienced	33 (40.2)	136 (81.9)		
**Do you engage in farming?**				
I farm	84 (97.7)	166 (96.5)	0.3	0.723
I do not farm	2 (2.3)	6 (3.5)		
**Water source for farming**				
Dam A	54 (64.3)	75 (45.2)	8.2	0.004*
Dam B	30 (35.7)	91 (54.8)		
**Do you swim in a dam?**				
I swim	72 (86.8)	46 (26.7)	81.1	<0.001*
I do not swim	11 (13.3)	126 (73.3)		
**Water source for swimming**				
Dam A	13 (20.3)	29 (37.2)	4.8	0.028*
Dam B	51(79.7)	49 (62.8)		
**Do you engage in fishing?**				
I fish	62 (72.1)	45 (26.2)	49.8	<0.001*
I do not fish	24 (27.9)	127 (73.8)		
**Water source for fishing**				
Dam A	9 (10.5)	129 (75.0)		<0.001*
Dam B	77 (89.5)	43 (25.0)		

Significant association of student characteristics and water activity with the occurrence of bloody urine

Bivariate analysis showed that sex of students, swimming, engaging in fishing and having experienced previous episodes of bloody urine were significantly associated with having a recent onset of bloody urine (Table 4). Students who swam in a dam had seventeen times more odds of urinating blood compared to those who did not swim (cOR=17.93, 95% CI=8.74-36.79). Likewise, students who practiced fishing practices were had seven times increased odds of acquiring schistosomiasis with colleagues who did not fish (cOR=7.29, 95% CI=4.08-13.04). The odds of cases who had been previously treated for episodes of haematuria was 6 times more compared to students without previous episodes of haematuria (cOR=6.73, 95% CI=3.72-12.17). Water-related activities in Dam B were found to have greater odds than same activities in Dam A; fishing (cOR=25.67, 95% CI=11.86-55.54), swimming (cOR=17.93, 95% CI=8.74-36.79), and farming (cOR=17.93, 95% CI=8.74-36.79) (Table 4).

**Table 4:** bivariate and multivariate analysis for risk factors among cases and controls at the basic schools

Variable	Bivariate analysis		Multivariate analysis	
	cOR (95% CI)	P value	aOR (95%CI)	P value
**Sex**				
Female	1	0.002*	1	0.401
Male	2.31 (1.3-3.96)		1. 60 (0.53-4.82)	
**Do you swim in a dam?**				
No	1	<0.001*	1	0.001*
Yes	17.93 (8.74-36.79)		44.63 (4.73-420.86)	
**Which Dam do you swim in?**				
Dam A	1	0.030*	1	0.279
Dam B	0.63 (0.20-0.92)		0.72 (0.03-2.78)	
**Do you engage in fishing?**				
No	1	<0.001*	1	0.780
Yes	7.29 (4.08-13.04)		1.23 (0.28-.39)	
**Which Dam do you fish in?**				
Dam A	1	<0.001*	1	<0.001*
Dam B	25.67 (11.86-55.54)		19.66 (4.21-91.76)	
**What dam do fetch for farming?**				
Dam A	1	0.005*	1	0.810
Dam B	2.18 (1.27-3.75)		0.77 (0.09-6.74)	
**Previous episode of bloody urine**				
No	1	<0.001*	1	0.014*
Yes	6.73 (3.72-12.17)		3.86 (1.31-11.39)	

* Multivariate variable with significant association of activity of students and their characteristics with the occurrence of bloody urine

After controlling for sex, swimming, fishing, previous episodes of haematuria, and the type of Dam students had activities in at multivariate level, swimming among students had 44 times greater odds compared to not swimming (aOR=44.63, 95% CI=4.73-420.86). Children who swam in dam B were 63% more likely to be cases compared to those who swam in dam A (aOR=0.63, 95% CI=0.20-0.92). Students who engaged in fishing practices in dam B had 19.66 times greater odds of infestation compared to fishing in dam A (aOR=19.66, 95% CI=4.21-91.76) at the multivariate level. The odds of having urogenital schistosomiasis among students who had been previously episodes of bloody urine was 3.86 times greater than the odds among children who had not experienced previous episodes (aOR=3.86, 95% CI=1.31-11.39) (Table 4).

## Discussion

We investigated an outbreak of Urogenital Schistosomiasis to determine its magnitude and risk factors and to implement control measures. Our investigation showed a widespread infection among students of the basic school. Swimming in dam B and having experienced a previous episode of bloody urine were independent risk factors for disease occurrence among the students.

The 5-year threshold analysis of bloody urine in the Ketu North District showed five potential outbreaks that were missed over the period. This was because the District Health Authority had not determined these thresholds to monitor the trend of routine data. However, the highest number of reported bloody urine cases was observed from the basic school in the period of this outbreak. There is a need for routine analysis of surveillance data to ensure that outbreaks are detected on time, and control measures put in place to prevent widespread infections. This requires building district level capacity for epidemiological expertise.

The months of May and August form the peak rainy season in Ghana. The maturation of snails occurs during this rainy season, and are able to transmit cercarie in the water bodies leading to greater infestation. A quantitative analysis of rainfall changes at the Ga District of Ghana showed a positive correlation between the prevalence of schistosomiasis and rainfall [2]. During this outbreak, the basic school vacated at the period where reproduction and transmission of cercariae were maximum. During vacation, students engaged in water-related activities in dams and supported their parents to fish and farm. This might have increased exposure to the infective agents. These factors together are likely responsible for the upsurge in cases among the students.

Up to two-thirds of cases were males who were found to be an at-risk group in this study. This is consistent with findings observed in Namibia [13], Ghana [6,12] and Nigeria [14]. Compared to girls, boys engage more in swimming and also participate in fishing and farming, which are significant risk factors for Schistosomiasis [6,9,8,15]. In the Central Region of Ghana, however, a previous study identified more cases were females [15]. Cultural differences in these environments may be the reason for difference in finding. Females in some regions perform most household chores like washing clothes in infested water bodies, and may make them more predisposed.

All suspected cases reported haematuria in our investigation. Persons with Schistosomiasis infection may not always present with haematuria, yet there remains a highly significant association between haematuria and schistosomiasis infection, making haematuria a useful symptom for case finding [11]. Thirty-nine percent of suspected cases also had bloody stools. *Schistosomiasis* outbreak in Mangwe District in Zimbabwe, had 9.5% of students with *haematuria* also reporting bloody stools [15]. Although about a quarter of the cases reported bloody stool, we could not ascertain whether the occurrence of bloody stool was a result of co-infection with *Schistosoma mansoni* or other intestinal worms. We did not test stool samples in this outbreak because of resource constraints. However, a cross-sectional prevalence study of Schistosomiasis at Kano State in Nigeria confirmed a *Schistosoma haematobium* and *mansoni* co-infection rate in the population at 0.5% [14]. The occurrence of Urogenital Schistosomiasis and other enteric parasites has also been documented in some studies among children [16]. It would have been helpful in our work if stool samples were tested. This remains an important area of further research within this affected population.

Having a previous episode of haematuria or a history of schistosomiasis treatment was an independent risk factor to having a recent infection. In Nigeria, persons with previous Urogenital Schistosomiasis were 2.87 times more likely to be re-infected [14]. This is because schistosomiasis is influenced by the knowledge and practices of persons at risk [17]. Individuals, who have had prior exposures, but have no improved knowledge and sanitation practices will still be predisposed to recurrent infections even after treatment. Improvement in sanitation and water supply is needed to complement knowledge and practices to protect at risk populations [18,19].

Between the two water bodies, we observed that dam B was a more likely source of infestation than dam A. Swimming and fishing in dam B, as well as irrigating farms with dam B were identified as risk factors for urogenital schistosomiasis amongst the students. Fishing practices in dam B was actually an independent risk to being infected with up to about 20 times increased odds compared to fishing in dam A. Dam B is the larger water source and is used by most of the students more frequently for swimming, fishing and farming purposes as compared to the dam A. These findings are also not surprising, as swimming, farming and fishing have been identified as risk factors for schistosomiasis in various studies [8,15]. Although the community has a borehole for water access, students preferred fetching water from the dams for chores. During these moments they get the opportunity to engage in recreational activities like swimming and several water contact activities. These predispose them to schistosomiasis infection.

As part of public health action, we conducted preventive measures including health education of students on WASH practices. We encouraged the use of borehole water within the communities instead of using water from the dam for domestic use. Mass drug administration with Praziquantel was done in the basic schools and communities in the district by standard protocol [20-22]. We recommended to the district political authorities and community leaders to set up prohibition notices to discourage recreational and domestic water activities by community members until disinfection of dams was done. In the light of resource limitation, we recommended that dam B was prioritized for disinfection. We also recommended further investigation of bloody stool among some students of the school.

**Limitations:** we recruited asymptomatic students as controls without testing them due to inadequate laboratory resources. This could have led to misclassification of asymptomatic cases as controls thereby affecting the strength of association between our studied exposures and our outcome. Additionally, we could not determine the frequency of exposure of cases and controls to the dams due to recall bias among respondents. However, research shows that a single exposure to infested water can lead to infestation [8].

## Conclusion

Schistosomiasis haematobium was identified as the causative agent of this outbreak of bloody urine among students of the basic school. Major at risk groups were male students, students who swam in dams, and engaged in fishing practices. Of the dams in the communities, dam B was a more likely source of the infection. WASH education, environmental control, and mass drug administration were key interventions implemented.

### What is known about this topic

- Urogenital Schistosomiasis occurs mostly among children of school going age;
- Water-related activities like swimming and fishing are significant associated factors to acquiring Urogenital Schistosomiasis.

### What this study adds

- Using a case-control study design, this study isolated one of two dams which was the likely source of the infection among affected students;
- Students previously treated for schistosomiasis infestation are still more likely to be affected in a new outbreak.
